# Positive surgical margins are predictors of local recurrence in conservative kidney surgery for pT1 tumors

**DOI:** 10.1590/S1677-5538.IBJU.2017.0039

**Published:** 2018

**Authors:** Patricio Garcia Marchiñena, Sebastián Tirapegui, Ignacio Tobia Gonzalez, Alberto Jurado, Guillermo Gueglio

**Affiliations:** 1 Department of Urology, Hospital Italiano de Buenos Aires, Buenos Aires, Argentina

**Keywords:** Kidney, Surgical Procedures, Neoplasms, Prognosis

## Abstract

**Objectives:**

The clinical significance of positive surgical margin (PSM) after a Nephron Sparing Surgery (NSS) is controversial. The aim of this study is to evaluate the association between PSM and the risk of disease recurrence in patients with pT1 kidney tumors who underwent NSS.

**Materials and Methods:**

Retrospective cohort study. A total of 314 patients submitted to a NSS due to stage pT1 renal tumor between January 2010 and June 2015 were included. Recurrence-free survival was estimated. The Cox model was used to adjust the tumor size, histological grade, pathological stage, age, surgical margins and type of approach.

**Results:**

Overall PSM was 6.3% (n=22). Recurrence was evidenced in 9.1% (n=2) of patients with PSM and 3.5% (n=10) for the group of negative surgical margin (NSM). The estimated local recurrence-free survival rate at 3 years was 96.4% (95% CI 91.9 to 100) for the NSM group and 87.8% (95% CI 71.9 to 100) for PSM group (p=0.02) with no difference in metastasis-free survival. The PSM and pathological high grade (Fuhrman grade III or IV) were independent predictors of local recurrence in the multivariate analysis (HR 12.9, 95%CI 1.8-94, p=0.011 / HR 38.3, 95%CI 3.1-467, p=0.004 respectively). Fuhrman grade proved to be predictor of distant recurrence (HR 8.1, 95%CI 1.6-39.7, p=0.011).

**Conclusions:**

The PSM in pT1 renal tumors showed to have higher risk of local recurrence and thus, worse oncological prognosis.

## INTRODUCTION

The increase in cross-sectional imaging studies in the last decades has made possible early detection of renal tumors. These achievements have propelled the Nephron Sparing Surgery (NSS) as the gold standard in the treatment of Stage T1 renal tumors (1-3). Such procedures do not imply in a significant increase in the postoperative morbidity with some discussions about the oncological results. Although EORTC group published in 2011 level 1 evidence against NSS in terms of survival, limitations of the study (recognized even by authors) and other publications showed comparable outcomes between radical and conservative renal surgeries (4-6).

Minimally invasive surgical techniques has changed the treatment paradigm of patients with RCC stage T1. Literature is contradictive concerning the rate of positive surgical margins (PSM) considering the surgical approach. While some authors suggest that Laparoscopic Partial Nephrectomy (LPN) and robot-assisted partial nephrectomy present higher rates of PSM in comparison to Open Partial Nephrectomy (OPN) (7) others deny such difference (8).

The aim of this study is to evaluate the association of PSM with local and distant recurrence and describe the clinical and pathological characteristics that can predict recurrence in those patients with PSM and clinical stage T1.

## MATERIALS AND METHODS

This is a retrospective cohort study including patients with pT1 renal cell carcinoma that underwent NSS either by open or minimally invasive surgery (laparoscopic or robot-assisted), from January 2010 to June 2015 at the Hospital Italiano de Buenos Aires, Argentina. Three possible surgical techniques were used based on tumor location: enucleation (resection of the tumor only); polar nephrectomy (resection of the entire pole including single or multiple lesions) or hemi-nephrectomy (half of the kidney was resected including single or multiple lesions). Enucleation was the first choice whenever possible (exophytic lesions without sinus involvement). Minimally invasive and open NSS technique have been previously described (9, 10). Data was collected prospectively from the electronic clinical history. Demographic data and the clinical pathological characteristics as well as the follow-up registry were tabulated: gender, body mass index (BMI), tumor size, approach, histologic sub-classification, Fuhrman grade, PSM and clinical situation at the last follow-up (no recurrence, local recurrence, distant recurrence or death by cancer). Pathology exam data was detailed according to the sixth edition of the American Joint Committee on Cancer (AJCC) TNM (11). The nuclear grade was informed based on the Fuhrman classification (12). A computed tomography (CT) scan of the chest, abdomen and pelvis with intravenous contrast was performed for staging the tumor.

The surgical approach was registered (open, minimally invasive). All the resected pieces were revised by the same pathologist experienced in oncological urology. A positive margin was assigned to the tumor cell that contacted with chinese ink.

The oncologic follow-up was performed according to the NCCN guidelines (13).

Local recurrence was defined as a tumor mass in the ipsilateral kidney over the resection bed of the same histological type of the original tumor.

Patients older than 18 years of age with single renal tumors pT1a and pT1b with a minimum follow-up of three months were included.

Patients with multiple and/or bilateral tumors, metastases at the time of diagnosis, benign tumors or hereditary renal tumors (Von Hippel Lindau), were excluded.

The presence of PSM or NSM was registered. Both populations were compared and the oncologic results were analysed according to local or distant recurrence.

### Statistical analysis

Continuous normally distributed variables are expressed by their mean and standard deviation. Not normally distributed variables are expressed as medians and their interquartile (IQR) ranges and categorical variables are expressed as n (%). To compare the continuous variables with normal distribution we used the T-test. In case of not normally distribution we utilized the Mann-Whitney test. To compare the categorical variables we used the Chi-square or Fisher test if the assumption for the first was not complied. For the estimation of disease-free survival (DFS) we used the method of Kaplan Meier expressed in estimated time and its confidence interval of 95% (95% CI). In the case of comparing subgroups, the log rank test was used. For the estimation of risk in the univariable or multivariable analysis, we used the Cox regression, expressing the hazard ratio (HR) and 95% CI. In Cox multivariable models, cases with not informed Fuhrman grade were dropped. To avoid collinearity with stage (more important for us), we did not include tumor diameter in multivariable analysis. Statistical significance was considered to be at p<0.05. Analyses were performed using SPSS 18.0^®^.

The present study was approved by the Institutional Ethics Committee after the protocol's revision and the procedures used according to international regulations (CEPI 3028).

## RESULTS

In the period between January 2010 and June 2015, 347 NSS surgeries were performed in patients with pT1. Of all the studied patients, 314 met the inclusion criteria. There were 22 PSM (9 open, 12 laparoscopic and 1 robot-assisted), the rate of PSM for open, laparoscopic and robotic assisted approach was 6.3%, 5% and 7.9%, respectively (p=0.673). Table-1 shows no difference in baseline characteristics between groups by margin status. The minimally invasive approach was used in 172 (54.8%) patients, 20 of which were operated with robotic assistance. Clear cell carcinoma was the most common histologic type followed by chromophobe and papillary with no statistical difference in PSM (6.1%, 11.1%, and 10% respectively, p=0.79). The median follow-up was 24 months (IQR 12-40).

**Table 1:** Baseline characteristics.

	Total	NSM	PSM	p
No. pts (%)	314	292 (93)	22 (7)	
Male (%)	218 (69.4)	202 (69.2)	16 (72.7)	0.72
Mean Age (SD)	58.3 (12)	58.2 (12.2)	58.9 (14.8)	0.81
Mean BMI (SD)	28.2 (4.8)	28.2 (4.7)	28.6 (4.8)	0.67
**Approach (%)**				**0.67**
Open	142 (45.2)	133 (45.5)	9 (40.9)	
Minimally Invasive	172 (54.8)	159 (54.5)	13 (59.1)	
Median tumor size mm (IQR)	29.7 (21-38)	30 (21.2-38)	27 (20-30)	0.51
**Histology (%)**				**0.92**
Clear-cell	245 (78)	230 (78.8)	15 (68.2)	
Chromophobe	45 (14.3)	40 (13.7)	5 (22.7)	
Papillary type I	10 (3.2)	9 (3.1)	1 (4.5)	
Papillary type II	10 (3.2)	9 (3.1)	1 (4.5)	
Other	4 (1.3)	4 (1.3)	0 (0)	
**Fuhrman (%)**				**0.62**
Non informed	49 (15.6)	44 (15.1)	5 (22.3)	
Grade I y II	258 (82.2)	242 (82.9)	16 (72.7)	
Grade III y IV	7 (2.2)	6 (2)	1 (4.5)	
**Tumor Stage (%)**				**0.82**
pT1a	266 (84.7)	247 (84.6)	19 (86.4)	
pT1b	48 (15.3)	45 (15.4)	3 (13.6)	

During follow-up, local recurrence rate was 1.9% (6 patients). In PSM group we found 2 (9.1%) local recurrences both with synchronic distant metastases. In the NSM group there were 4 local recurrences (1.4%), 2 with synchronic distant metastases. Six patients, all in the NSM group, presented with distant metastases alone.

**Figure 1:**
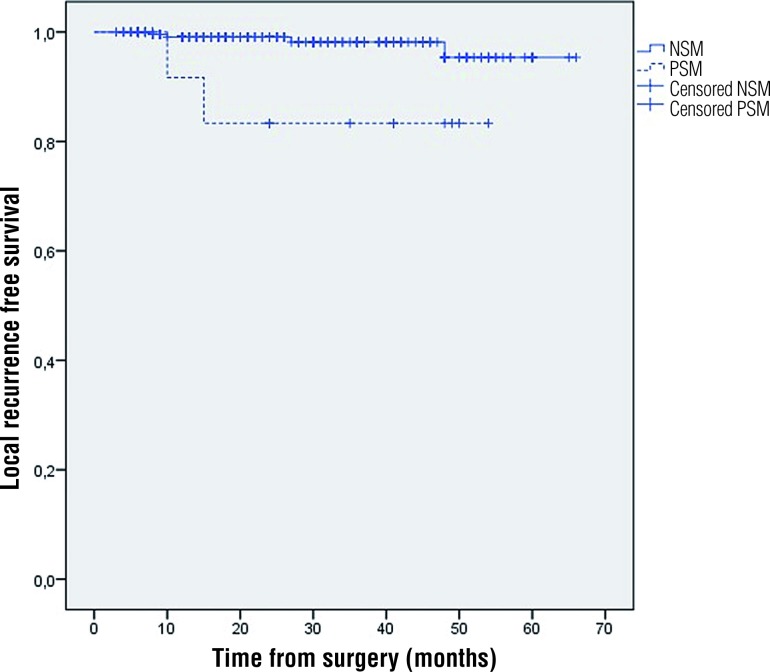
Local recurrence free survival by margin status.

The estimated local recurrence-free survival (LRFS) rate at 3 years for the NSM group was 96.4% (CI 91.9-100), while for the PSM group was 87.8% (95% CI 71.9-100), with a statistically significant difference (log rank test p=0.02; Figure-1). The estimated metastasis-free survival (MFS) rate at 3 years in patients with PSM was 87.8% (95% CI 71.9-100) and for those who had NSM was 95.4% (95% CI .92, 1-98.7), with no significant difference (p=0.127 log rank test). Only 1 patient died of disease progression at 12 months (pT1b, Fuhrman II, NSM).

The estimated 3 year LRFS and MFS for Fuhrman III-IV was 66.7% and 50% respectively, in contrast to 98.1% and 95.2% in Fuhrman I-II (p=0.0001).

PSM and Fuhrman grade III or IV were independent predictors of LRFS, while stage, age, tumor size and type of approach didn't result as predictors (Table-2). High Fuhrman grade and tumor stage were the only factors that increased the risk of metastases (Table-3). For multivariable analysis, cases without Fuhrman informed (i.e., chromophobe) were dropped out, thus cohort analysed included 265 patients.

**Table 2:** Univariable and multivariable analysis of local recurrence.

		Univariable		Multivariable	
		HR (95% CI)	p	HR (95% CI)	p
**PSM**		7.3 (1.33-40.2)	0.023	7.7 (1.3-47)	0.026
**Age**		1.01 (0.94-1.08)	0.718	1.02 (0.9-1.1)	0.601
**Tumor size mm**		1.01 (0.94-1.08)	0.701	++	++
**Fuhrman**					
	Grade I-II	Ref.		Ref.	
	Grade III-IV	20.3 (3.7-110)	0.001	16.3 (1.9-135)	0.01
**Stage**					
	pT1a	Ref.		Ref.	
	pT1b	2.5 (0.47-13.9)	0.28	0.8 (0.1-7.7)	0.861
**Approach**					
	Laparoscopic	Ref.		Ref.	
	Open	0.4 (0.08-2.5)	0.36	0.5 (0.08-4.1)	0.578

++ Excluded to avoid collinearity with Stage.

**Table 3:** Univariable and multivariable analysis of distant recurrence.

		Univariable		Multivariable	
		HR (95% CI)	p	HR (95% CI)	p
**PSM**		3.7 (0.8-17.7)	0.09	3.5 (0.7-17)	0.127
**Age**		1.03 (0.9-1.1)	0.305	1.03 (0.9-1.1)	0.266
**Tumor size mm**		1.08 (1.04-1.14)	0.0001	++	++
**Fuhrman**					
	Grade I-II	Ref.		Ref	
	Grade III-IV	19.5 (5-75.8)	0.001	9.4 (2.1-43)	0.004
**Stage**					
	pT1a	Ref.		Ref.	
	pT1b	8.23 (2.3-29.2)	0.001	5.2 (1.2-22)	0.026
**Approach**					
	Laparoscopic	Ref.		Ref.	
	Open	0.35 (0.09-1.3)	0.129	0.9 (0.2-4.4)	0.902

++ Excluded to avoid collinearity with Stage

The presence of local recurrence in the univari able analysis shows an increased risk of distant recurrence with a HR 33.4 (95% CI 9.3119, p=0.0001).

The PSM rate for group Fuhrman I-II and III-IV was 6.2% and 14.3%, respectively with no positive association (p=0.367).

## DISCUSSION

Trends in NSS are rising around the world, although there are some concerns about the sig-nificance of PSM in terms of oncological outcomes (14, 15). Nowadays incidental renal tumors are frequently found and suitable for NSS (16, 17). In this study, we evaluated the association between PSM and local or distant recurrence in patients with pT1 renal tumors undergoing NSS.

Several studies highlight the impact of PSM on recurrence free survival (14, 15, 18-27). Shah et al. recently reported in a large retrospective cohort of patients undergoing partial nephrectomy for localized renal tumors, that some patients with high risk disease (eg pT2-pT3a or Fuhrman grade II-III) with PSM will experience relapse with distant metastasis (28). As well as some authors (22, 28) we found that PSM was independently associated with local recurrence, but not directly linked with distant recurrence. This relationship was observed in higher Fuhrman grade (III and IV), but we didn't demonstrated increased risk in T1b tumors (over T1a). We didn't analyse histological type because our high prevalence of clear cell histology.

A multicentric retrospective study comparing 111 patients with PSM and 664 patients with NSM, concluded that patients with PSM presented a higher risk of local recurrence, even though there were no statistically significant differences in recurrence-free survival, cancer-specific sur-vival and overall survival (14). In relation to this work, the Memorial Sloan Kettering Cancer Center (20) analysed 777 PN between 1989 and 2005. Of 75 patients who had PSM (7.5%), only 2 developed local recurrence (4%), while from 713 patients (92; 5%) with NSM, 4 presented recurrence (0.5%). They concluded that patients with PSM have a higher incidence of local recurrence, not determined by the margin status. A bibliographic review of 3.803 RCC and 173 PSM concluded that a positive margin in PN rarely correlates with local recurrence (15, 20-23). Khalifeh et al. (24) published a total of 943 robot-assisted PNs; 21 patients (2.2%) had PSM and 922 had NSM. When comparing both groups, they concluded that PSM had higher rates of local recurrence and metastases (p<0.001) in relation to NSM and that there was a statistically significant difference in the recurrence-free survival and metastasis between the two groups (log rank test <0.001). PSM presented 18.4 more times the risk for recurrence in the multivariate study. Patients with PSM present worse oncological prognosis, higher probabilities of local recurrence of the disease (log rank test p=0.02) and perhaps more likely to have distant re-currence (although without significant difference, log rank test p=0.127). An interesting fact is that local recurrence increases the risk of metastasis (HR 33.4), which highlights the importance of determining the risk of local recurrence itself.

It is debatable to analyse recurrence without inform cancer specific survival that is the principal global oncologic outcome, even with PSM. Reports that underestimate the value of PSM, may incur in a bias in the truly effect of PSM in cancer survival.

It is controversial what to do with PSM. Although the American Urological Association and the National Comprehensive Cancer Network guidelines do not recognize the clinical significance of PSM, in view of our results it seems reasonable to follow-up all patients but with close surveillance imaging (28). Nephrectomy or repeat resection remains unnecessary with no study demonstrating clear advantages. Several studies observed that PSM not necessarily signify residual disease. Benalash et al. observed residual disease in only 39% of patients with PSM who underwent repeat surgery (14).

In agreement with other authors, we found that the rate of positive margins is comparable between open and minimally invasive surgery, for the path does not predispose to risk of recurrence (27, 29).

Although we have not routinely performed intra-operative frozen sections, its relevance grows as a PSM seems to impact in local relapse. Unfortunately, many authors suggest that intraoperative frozen section has limited utility. Arguments against the routine use of frozen section in NSS, are that it has a relatively high false-negative rate and inconsistency in changing intra-operative management (30).

This study presents some limitations. It is a retrospective study of a single center, so the results could be sensitive to selection bias. The low incidence of positive surgical margins and the relative infrequency of pathologically aggressive lesions treated with NSS explain the low number of events that lead us to wide HR confidence intervals that may limit the interpretations of our findings, despite significance, so caution must be taken in the results interpretation. On the other hand, finding this level of increased risk with this number of events is unlikely to happen by chance bias.

Based on the outcomes of uni/multivariable analysis and Kaplan Meier curves adjusted by grade and PSM, recurrences are more likely to happen in the first two years and not proportional with time, but follow-up is too short to ensure the proportional model.

## CONCLUSIONS

PSM in NSS for pT1 tumors has a lower local recurrence-free estimated survival compared to patients with NSM. Fuhrman grade is another predictor of disease free survival. The relationship between PSM and Fuhrman grade should be studied to ensure that the complete resection of the tumor should not be underestimated. However, larger series of patients and longer follow-up time is needed to draw more accurate conclusions.

## ETHICAL APPROVAL

All procedures performed in studies involving human participants were in accordance with the ethical standards of the institutional and/or national research committee and with the 1964 Helsinki declaration and its later amendments or comparable ethical standards. For this type of study, formal consent is not required.

## References

[1] Doeuk N, Guo DY, Haddad R, Lau H, Woo HH, Bariol S (2011). Renal cell carcinoma: stage, grade and histology migration over the last 15 years in a large Australian surgical series. BJU Int..

[2] Baillargeon-Gagné S, Jeldres C, Lughezzani G, Sun M, Isbarn H, Capitanio U (2010). A comparative population-based analysis of the rate of partial vs radical nephrectomy for clinically localized renal cell carcinoma. BJU Int..

[3] Zini L, Patard JJ, Capitanio U, Mejean A, Villers A, de La Taille A (2009). The use of partial nephrectomy in European tertiary care centers. Eur J Surg Oncol..

[4] Thompson RH, Siddiqui S, Lohse CM, Leibovich BC, Russo P, Blute ML (2009). Partial ver-sus radical nephrectomy for 4 to 7 cm renal cortical tumors. J Urol..

[5] Pahernik S, Roos F, Hampel C, Gillitzer R, Melchior SW, Thüroff JW (2006). Nephron sparing surgery for renal cell carcinoma with normal contralateral kidney: 25 years of ex-perience. J Urol..

[6] Van Poppel H, Da Pozzo L, Albrecht W, Matveev V, Bono A, Borkowski A (2011). A prospective, randomised EORTC intergroup phase 3 study comparing the oncologic outcome of elective nephron-sparing surgery and radical nephrectomy for low-stage renal cell carcinoma. Eur Urol..

[7] Tabayoyong W, Abouassaly R, Kiechle JE, Cherullo EE, Meropol NJ, Shah ND (2015). Variation in Surgical Margin Status by Surgical Approach among Patients Undergoing Partial Nephrectomy for Small Renal Masses. J Urol..

[8] Zargar H, Larson J, Ball MW (2014). Comparison of perioperative outcomes of robotic partial nephrectomy and open partial nephrectomy in in patients with solitary kidneys. J Urol..

[9] Costabel JI, Marchinena PG, Tirapegui F, Dantur A, Jurado A, Gueglio G (2016). Functional and oncologic outcomes after nephron-sparing surgery in a solitary kidney: 10 years of experience. Int Braz J Urol..

[10] Gueglio G, Jurado A, Tobía González, González MS, García Freire F, Liyo J (2008). Enucleation versus partial nephrectomy in the treatment of renal cell carcinoma. Rev Argent Urol..

[11] Greene FL, Page DL, Fleming ID (2002). AJCC cancer staging manual.

[12] Fuhrman SA, Lasky LC, Limas C (1982). Prognostic significance of morphologic parameters in renal cell carcinoma. Am J Surg Pathol..

[13] Motzer RJ, Jonasch E, Agarwal N, Beard C, Bhayani S, Bolger GB (2015). Kidney cancer, version 3.2015. J Natl Compr Canc Netw..

[14] Bensalah K, Pantuck AJ, Rioux-Leclercq N, Thuret R, Montorsi F, Karakiewicz PI (2010). Positive surgical margin appears to have negligible impact on survival of renal cell carcinomas treated by nephron-sparing surgery. Eur Urol..

[15] Permpongkosol S, Colombo JR, Gill IS, Kavoussi LR (2006). Positive surgical parenchymal margin after laparoscopic partial nephrectomy for renal cell carcinoma: oncological outcomes. J Urol..

[16] Dulabon LM, Lowrance WT, Russo P, Huang WC (2010). Trends in renal tumor surgery deli-very within the United States. Cancer..

[17] Patel HD, Mullins JK, Pierorazio PM, Jayram G, Cohen JE, Matlaga BR (2013). Trends in renal surgery: robotic technology is associated with increased use of partial nephrec-tomy. J Urol..

[18] Carini M, Minervini A, Masieri L, Lapini A, Serni S (2006). Simple enucleation for the treat-ment of PT1a renal cell carcinoma: our 20-year experience. Eur Urol..

[19] Sundaram V, Figenshau RS, Roytman TM, Kibel AS, Grubb RL, Bullock A (2011). Positive margin during partial nephrectomy: does câncer remain in the renal remnant?. Urology..

[20] Kwon EO, Carver BS, Snyder ME, Russo P (2007). Impact of positive surgical margins in pa-tients undergoing partial nephrectomy for renal cortical tumours. BJU Int..

[21] Piper NY, Bishoff JT, Magee C, Haffron JM, Flanigan RC, Mintiens A (2001). Is a 1-CM margin necessary during nephron-sparing surgery for renal cell carcinoma?. Urology..

[22] Sutherland SE, Resnick MI, Maclennan GT, Goldman HB (2002). Does the size of the surgical margin in partial nephrectomy for renal cell cancer really matter?. J Urol..

[23] Duvdevani M, Laufer M, Kastin A, Mor Y, Nadu A, Hanani J (2005). Is frozen section analysis in nephron sparing surgery necessary? A clinicopathological study of 301 cases. J Urol..

[24] Khalifeh A, Kaouk JH, Bhayani S, Rogers C, Stifelman M, Tanagho YS (2013). Positive surgical margins in robot-assisted partial nephrectomy: a multi-institutional analysis of oncologic outcomes (leave no tumor behind). J Urol..

[25] Minervini A, di Cristofano C, Lapini A, Marchi M, Lanzi F, Giubilei G (2009). Histo-pathologic analysis of peritumoral pseudocapsule and surgical margin status after tumor enucleation for renal cell carcinoma. Eur Urol..

[26] Permpongkosol S, Colombo JR, Gill IS, Kavoussi LR (2006). Positive surgical parenchymal margin after laparoscopic partial nephrectomy for renal cell carcinoma: oncological outcomes. J Urol..

[27] Breda A, Stepanian SV, Liao J, Lam JS, Guazzoni G, Stifelman M (2007). Positive mar-gins in laparoscopic partial nephrectomy in 855 cases: a multi-institutional survey from the United States and Europe. J Urol..

[28] Shah PH, Moreira DM, Okhunov Z, Patel VR, Chopra S, Razmaria AA (2016). Positive Surgical Margins Increase Risk of Recurrence after Partial Nephrectomy for High Risk Renal Tumors. J Urol..

[29] Marszalek M, Meixl H, Polajnar M, Rauchenwald M, Jeschke K, Madersbacher S (2009). La-paroscopic and open partial nephrectomy: a matched-pair comparison of 200 patients. Eur Urol..

[30] Gordetsky J, Gorin MA, Canner J, Ball MW, Pierorazio PM, Allaf ME (2015). Frozen section during partial nephrectomy: does it predict positive margins?. BJU Int..

